# Translation and Validation of the Thai Version of the Telephone Interview for Cognitive Status and the Mini Montreal Cognitive Assessment in Older Adults

**DOI:** 10.1002/brb3.71178

**Published:** 2026-01-07

**Authors:** Pasa Sukson, Weerasak Muangpaisan, Supakorn Chansaengpetch, Angkana Jongsawadipatana, Pitiporn Siritipakorn, Ananya Treewisut, Jirawit Wong‐ekkabut, Somboon Intalapaporn

**Affiliations:** ^1^ Department of Medicine, Faculty of Medicine Siriraj Hospital Mahidol University Bangkok Thailand; ^2^ Department of Preventive and Social Medicine, Faculty of Medicine Siriraj Hospital Mahidol University Bangkok Thailand; ^3^ Department of Nursing, Faculty of Medicine Siriraj Hospital Mahidol University Bangkok Thailand

**Keywords:** dementia, MCI, Mini MoCA, TICS

## Abstract

**Introduction:**

This study aimed to translate and validate the telephone interview for cognitive status (TICS) and the mini montreal cognitive assessment (Mini MoCA) for use in older Thai adults and to compare their diagnostic validity for mild cognitive impairment (MCI) and dementia.

**Materials and methods:**

A total of 149 participants—51 cognitively normal (CN), 49 with MCI, and 49 with dementia—were enrolled. Diagnoses were based on DSM‐5 criteria and the Clinical Dementia Rating (CDR), determined by a senior geriatric neurologist. Participants also completed the MMSE‐2, MoCA, NPI‐Q, and ADL assessments, administered by certified psychologists and a geriatric nurse. The TICS and Mini MoCA were administered by two independent, blinded clinicians within four weeks of the initial evaluation. Test‐retest reliability was assessed after two weeks. Validity and reliability analyses included content and construct validity, and inter‐rater/test‐retest reliability.

**Results:**

Mean age and education were comparable across groups. Both TICS and Mini MoCA scores correlated significantly with standard cognitive and functional measures. Test‐retest and inter‐rater reliability were excellent (ICC = 0.933 and 0.995 for TICS; 0.918 and 0.998 for Mini MoCA). For discriminating CN from dementia, sensitivity/specificity were 81.3%/81.2% (AUC = 0.883) for TICS and 87.5%/89.6% (AUC = 0.958) for Mini MoCA. For CN vs. MCI, Mini MoCA (AUC = 0.755) performed slightly better than TICS (AUC = 0.693). Average administration times were 8.7 and 4.4 min, respectively.

**Conclusions:**

TICS and Mini MoCA are valid, reliable tools for cognitive screening in older Thai adults, with Mini MoCA showing slightly superior performance.

## Abstract 2

Mini MoCA‐Thai and TICS‐Thai, culturally adapted for older adults, showed high reliability and strong validity. Mini MoCA‐Thai performed slightly better for detecting MCI and dementia, and both tools are practical for brief, phone‐based cognitive screening in Thai community settings.

## 1 Introduction

Significant progress has been made in developing cognitive screening tools aimed at early detection of cognitive decline (Prince et al. 2015; Kanjanapong et al. 2021). Among the most widely used instruments are the mini‐mental state examination (MMSE) (Folstein et al. 1975) and the MoCA (Nasreddine et al. 2005), which demonstrate strong sensitivity and specificity for identifying dementia and MCI, respectively. However, both tools have inherent limitations. They require face‐to‐face administration, involve relatively lengthy testing durations, and may not be feasible for individuals residing in remote areas or those with limited mobility. Furthermore, their reliance on visual or motor responses makes them less suitable for individuals with visual impairments or certain physical disabilities.

To overcome these challenges, telephone‐based cognitive assessments have been increasingly adopted as viable alternatives for cognitive screening (Castanho et al. 2014; McDicken et al. 2019; Pendlebury et al. 2013). These tools offer several advantages, including shorter administration time, cost‐effectiveness, and the ability to reach populations without access to advanced technologies or healthcare facilities. Their utility became even more apparent during the COVID‐19 pandemic, as they facilitated remote assessments while maintaining infection control through physical distancing. Nevertheless, telephone‐based assessments present their own challenges, such as reduced applicability in individuals with hearing impairments, susceptibility to fraud, and potential variability in call quality that may influence test administration and results (Castanho et al. 2014; Pendlebury et al. 2013; Zietemann et al. 2017).

The TICS (Brandt et al. 1988) is one such tool developed as a telephone‐adapted alternative to the MMSE. It comprises 11 items assessing a range of cognitive domains, including orientation, attention, memory, repetition, conceptual knowledge, and nonverbal praxis, with a maximum score of 41. TICS has been translated and validated in several languages—such as Hebrew (Beeri et al. 2003), Italian (Dal Forno et al. 2006), Japanese (Konagaya et al. 2007), French (Vercambre et al. 2010), Korean (Seo et al. 2011), German (Matrisch et al. 2012), Portuguese (Baccaro et al. 2015), Greek (Georgakis et al. 2017), and Spanish (Munoz‐Garcia et al. 2019)—and has demonstrated high reliability and diagnostic accuracy in detecting cognitive impairment. Key strengths of the TICS include its brief administration time (approximately 10 min), strong psychometric properties, and suitability for remote cognitive screening. Despite its widespread use globally, the TICS has not been adapted and validated in the Thai language, where cultural, linguistic, and educational factors may influence performance and interpretation. Therefore, it is essential to translate and validate a Thai version of TICS to enhance its applicability in older Thai adults.

The Mini MoCA (MoCA Test Inc n.d.) is a brief, telephone‐administered cognitive screening instrument derived from the original MoCA, designed to provide a rapid (approximately 5 min) assessment of key cognitive domains. (Granier and Segal 2023) It includes four core items, yielding a total score of 15, and evaluates cognitive domains such as attention, executive function, memory, language, and orientation. Official instructions and protocols used in the present study were obtained directly from the MoCA's official website (MoCA Test Inc n.d.).

Notably, there are other versions of the Mini MoCA/MoCA‐5 developed by other authors that include different scoring systems, including a version derived from the Hong Kong MoCA (Wong et al. 2015), with a maximum score of 30 and the Dong MoCA (Dong et al. 2016), which has a maximum score of 12. The Mini MoCA has been translated into French (Dujardin et al. 2021) and Mandarin Chinese (Feng et al. 2021), with validation studies demonstrating good diagnostic performance for detecting dementia, with reported sensitivity and specificity of 87.3% and 76.1%, respectively (Dujardin et al. 2021). Its brevity—requiring approximately five minutes for administration—makes it particularly well‐suited for clinical or community settings where time constraints and resource limitations exist. However, evidence regarding its use in identifying MCI remains limited, and its cross‐cultural applicability, including in Thai populations, has not been systematically evaluated (Wong et al. 2015; Dong et al. 2016; Dujardin et al. 2021; Feng et al. 2021).

The primary objectives of this study were to (1) translate the TICS into Thai; and (2) validate both the Thai versions of the TICS and Mini MoCA in older Thai adults for distinguishing among individuals with dementia, MCI, and those who are CN. Secondary objectives included: (1) evaluating the agreement between TICS‐Thai and MMSE‐2, as well as Mini MoCA‐Thai and MoCA; (2) assessing the test‐retest reliability of both instruments; (3) determining inter‐rater reliability; and (4) comparing the diagnostic performance of TICS‐Thai and Mini MoCA‐Thai in the evaluation of cognitive status.

## 2 Materials and Methods

### 2.1 Study Design

This study employed a cross‐sectional validation design to translate and validate the Thai versions of the TICS and the Mini MoCA for use in older adults. Participants were recruited from the geriatric clinic at Siriraj Hospital, Mahidol University.

The inclusion criteria were as follows:

1. Adults aged 60 years and older attending outpatient visits at the geriatric clinic.
2. Individuals diagnosed with dementia or MCI according to the diagnostic and statistical manual of mental disorders, fifth edition (DSM‐5) (American Psychiatric Association 2013) criteria and the CDR scale (Morris 1993);
3. Individuals classified as CN by clinical interview .nd expert consensus.
4. Ability to communicate in and comprehend the Thai language.

All participants enrolled in this study were of Thai ethnicity and native Thai speakers. Although the inclusion criteria specified the ability to communicate in and comprehend the Thai language, all individuals included in the study were fully fluent and used Thai as their primary language in daily life. This linguistic and ethnic homogeneity supports internal validity.

The exclusion criteria included:

1. Presence of psychiatric or neurological conditions that could affect cognitive assessment, such as major depressive disorder or severe behavioral and psychological symptoms of dementia (BPSD) that impaired communication.
2. Diagnosis of moderate to severe dementia (CDR > 1) (Morris 1993).
3. Presence of acute medical conditions that could interfere with participation, such as recent hospitalization within the past three months.
4. Current use of substances or medications known to impair cognition, including alcohol, benzodiazepines, anticholinergic agents, or other addictive substances.
5. Hearing impairments that would interfere with telephone‐based assessments.
6. Physical disabilities that would hinder telephone assessment, motor response (e.g., tapping), or verbal communication, such as advanced Parkinson's disease or cerebrovascular disorders.
7. Refusal of informed consent by the participant or lack of consent from caregivers or legal representatives for participation in CDR assessments.

Withdrawal criteria included:

1. Voluntary withdrawal by the participant or their legal representative at any time during the study.
2. Failure to comply with telephone‐based assessments on two occasions (or three occasions in cases of test‐retest reliability evaluation).

### 2.2 Translations and Cross‐cultural Adaptation

The Mini MoCA (MoCA Test Inc., n.d.) had previously been translated into Thai. For the current study, the research team obtained formal permission and a licensing agreement from Dr. Ziad Nasreddine and MoCA to utilize the Mini MoCA‐Thai, which has a maximum total score of 15.

For the TICS (Brandt et al. 1988), the researchers contacted Dr. Jason Brandt, the original developer of the instrument, as well as the publisher, psychological assessment resources (PAR), to request permission and a licensing agreement for translation and use in this study. The translation and cross‐cultural adaptation process followed the standardized methodology recommended by Beaton et al. (Beaton et al. 2000) The forward translation was independently conducted by one of the authors, and a preliminary Thai draft was created through consensus by the study team. This version was subsequently reviewed by bilingual experts who conducted an independent back‐translation into English. Both the forward and back‐translated versions were submitted to the senior permissions specialist at PAR for final review and approval.

The Thai version of the TICS maintained the overall structure and content of the original instrument, with minor cultural adaptations approved by PAR to ensure contextual relevance. For example, the translation of the full name item (item 1) and the repetition task (item 8) was modified to suit Thai linguistic and cultural norms. Additionally, item 9, which originally referenced the “president” and “vice president,” was modified to “prime minister” and “deputy prime minister,” respectively, to reflect Thailand's political system. The maximum total score for the TICS‐Thai is 41.

The content validity of the TICS‐Thai was evaluated using the item‐level content validity index (I‐CVI). The assessment was conducted by a panel of four experts, including two geriatricians, one psychiatrist, and one Ph.D.‐level nursing specialist in gerontology from the geriatric clinic at our institution. The resulting I‐CVI was 1.0, indicating excellent content validity.

A pilot version of the TICS‐Thai was tested among 30 non‐clinical participants, comprising 10 older adult patients, 10 caregivers, and 10 healthcare personnel from the Geriatric Clinic at Siriraj hospital, Mahidol University. Participants were asked to provide feedback on the clarity, comprehensibility, and cultural appropriateness of the questionnaire using a 4‐point Likert scale (1 = completely agree, 2 = moderately agree, 3 = mildly agree, 4 = disagree). The mean scores for clarity, comprehensibility, and cultural appropriateness ranged from 1.00 to 1.53, 1.00 to 1.57, and 1.00 to 1.47, respectively, suggesting a high degree of acceptability and cultural relevance.

### 2.3 Validation Process of the Thai Version

Older adults attending outpatient visits at the geriatric clinic, Siriraj hospital, were recruited using simple random sampling. Preliminary eligibility screening was conducted using a standardized checklist. Informed consent was obtained from each participant or their legal representative following a detailed explanation of the study procedures.

Baseline demographic and clinical information was collected, including name, gender, age, occupation, education level, current address, primary caregiver, underlying medical conditions, current medications, history of alcohol use, hearing impairment, psychiatric history, and neurological conditions affecting cognition (e.g., depression).

Each participant underwent standardized assessments administered by trained clinical psychologists, including the MMSE, second edition (MMSE‐2 Thai) (Institute of Geriatric Medicine. MMSE‐Thai Version 2002; Folstein et al. 2010), the MoCA‐Thai (Nasreddine et al. 2005), the CDR (Morris 1993), and the neuropsychiatric inventory questionnaire (NPI‐Q Thai) (Kaufer et al. 2000, Hemrungrojn 2011). The diagnosis of MCI or dementia was made by an expert geriatric neurologist in accordance with the *DSM‐5* criteria (American Psychiatric Association 2013) and CDR scores (Morris 1993), based on a comprehensive evaluation that included clinical history, neurological examination, neuroimaging (CT or MRI of the brain), and findings from a comprehensive geriatric assessment (CGA).

**FIGURE 1:**
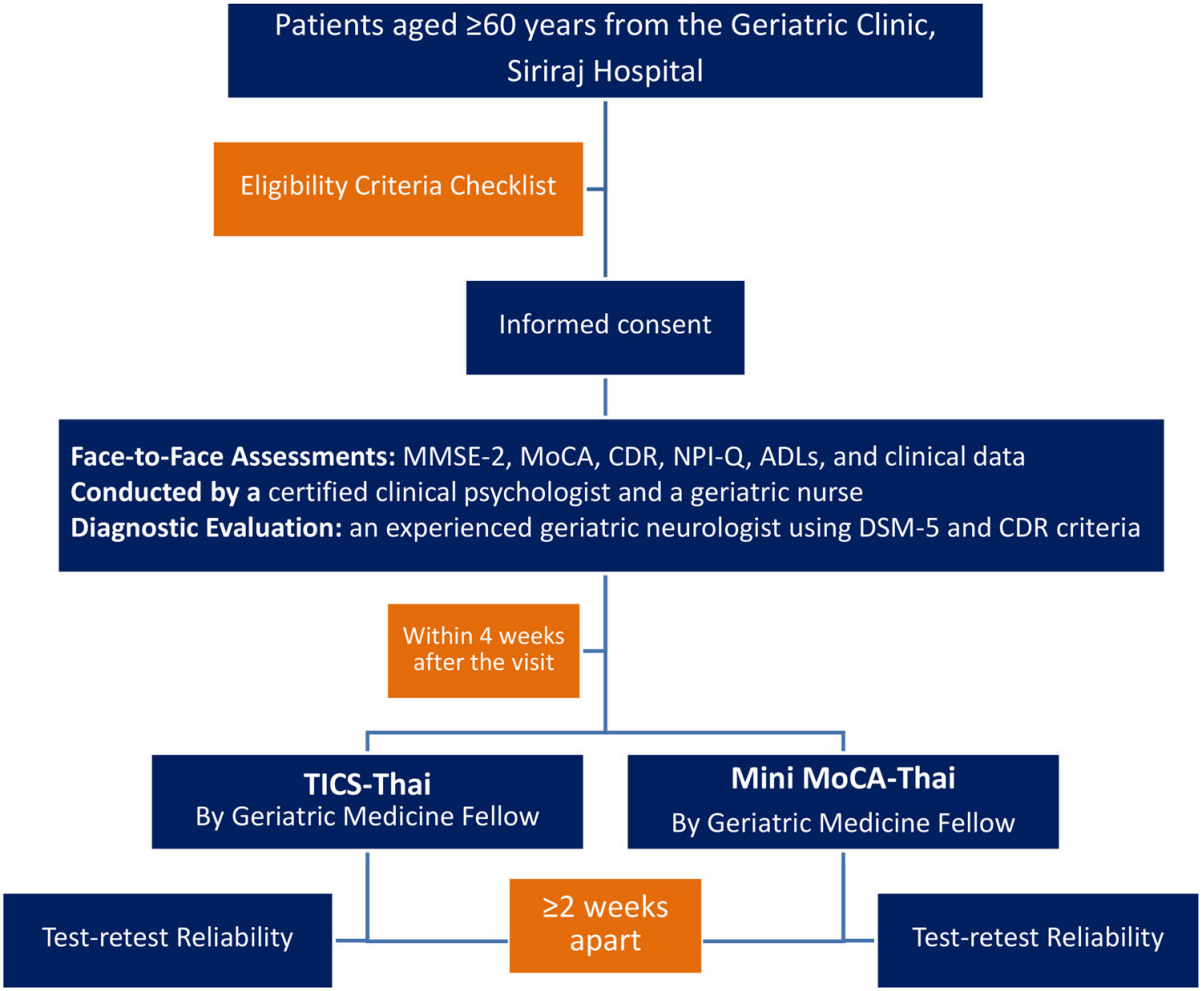
Flow of the study.

Within four weeks of the baseline evaluation, participants completed both the TICS‐Thai and Mini MoCA‐Thai, with each tool administered at least one week apart to minimize recall bias. To minimize the potential for order effects and practice effects when administering two cognitive assessments. To address this, we randomized participants into two groups: one group completed the TICS‐Thai first followed by the Mini MoCA‐Thai, while the other group completed the Mini MoCA‐Thai first. This simple randomization procedure was implemented specifically to counterbalance administration order and minimize any bias related to learning or exposure. For test‐retest reliability, a subset of participants underwent a second telephone assessment at a minimum interval of two weeks. The researcher conducting the telephone interviews was blinded to the participants’ clinical diagnoses. All data were collected, coded, and entered into a secure database for subsequent statistical analysis. Figure 1 shows the flow of the study.

### 2.4 Ethical Consideration

The research protocol and all study procedures were approved by the Siriraj Institutional Review Board, Faculty of Medicine Siriraj Hospital, Mahidol University, Bangkok, Thailand. Written informed consent was obtained from all participants or their legal representatives prior to enrollment.

### 2.5 Statistical Analysis

We performed comprehensive statistical analyses to evaluate the diagnostic performance of the TICS‐Thai and Mini MoCA‐Thai. For continuous variables, data were summarized using means and standard deviations (mean ± SD), while the median was reported for ordinal variables such as the CDR. Pearson's correlation coefficients were calculated to examine the relationships between TICS‐Thai, Mini MoCA‐Thai, and other cognitive assessment scores. The intra‐class correlation coefficient (ICC) was used to assess both test‐retest reliability and inter‐rater reliability of the TICS‐Thai and Mini MoCA‐Thai.

To evaluate concurrent validity in identifying dementia and MCI, receiver operating characteristic (ROC) curve analyses were conducted. The area under the curve (AUC) was calculated to determine the discriminative ability of each instrument for distinguishing individuals with dementia and MCI from CN participants. Additionally, we calculated the sensitivity, specificity, positive predictive value (PPV), negative predictive value (NPV), positive likelihood ratio (+LR), and negative likelihood ratio (−LR) for both tools to determine optimal cut‐off scores. These diagnostic parameters were used to assess the accuracy and clinical utility of the TICS‐Thai and Mini MoCA‐Thai in detecting cognitive impairment.

### 2.6 Sample Size Determination

Participants were categorized into three diagnostic groups (dementia, MCI, and CN). Sample size requirements were guided by two methodological considerations. First, diagnostic accuracy literature indicates that estimating sensitivity or specificity with a 95% confidence level and a margin of error of 0.10 requires approximately 40–45 participants per group (Flahault et al. 2005; Buderer 1996), which aligns with reported sensitivities and specificities of 0.70–0.90 for telephone‐based screening tools.

Second, psychometric guidelines such as COSMIN recommend recruiting at least 5–10 participants per item for reliability and validity assessment. (Mokkink et al. 2010; Anthoine et al. 2014) Based on the 11 items of the TICS‐Thai and the four items of the Mini MoCA‐Thai, a total sample of 120–150 participants was considered adequate to ensure stable psychometric estimates.

Accordingly, we aimed to recruit about 50 participants per diagnostic group. The final sample comprised 149 participants (51 CN, 49 MCI, 49 dementia), meeting and exceeding the minimum requirements derived from both diagnostic and psychometric frameworks. This sample size is consistent with recommended minimums to ensure acceptable precision and adequate statistical power for estimating diagnostic accuracy and reliability parameters.

## 3 Results

### 3.1 Participant Characteristics

A total of 149 participants from the geriatric clinic were enrolled in this study between 2022 and 2024. The cohort consisted of 51 CN older adults, 49 individuals with MCI, and 49 individuals with dementia. The mean ages of the CN, MCI, and dementia groups were 76.2 ± 4.7, 75.6 ± 4.8, and 77.0 ± 4.5 years, respectively, while the mean years of education were 12.4 ± 5.5, 11.1 ± 5.3, and 11.7 ± 5.1 years, respectively. No statistically significant differences in age or education were observed among the groups. Demographic and clinical characteristics are summarized in Table 1.

**TABLE 1:** Demographic and clinical characteristics of study participants (*n* = 149).

Parameter (ranges)	CN (*n* = 51)	MCI (*n* = 49)	Dementia (*n* = 49)	
	Mean ± SD			
Age, years (65–87)	76.2 ± 4.7	75.6 ± 4.8		77.0 ± 4.5
Women, *n* (%)	35 (68.6)	35 (71.4)		34 (69.4)
Education, years (0–18)	12.5 ± 5.5	11.1 ± 5.3		11.7 ± 5.1
Barthel index (11–20)	19.8 ± 0.6	19.7 ± 0.9		19.2 ± 1.9
Lawton IADL (2–12)	11.6 ± 1.4	11.2 ± 1.4		7.8 ± 3.1
MMSE‐2 (10–30)	24.6 ± 3.2	22.7 ± 3.3		17.3 ± 3.7
MoCA (5–29)	21.9 ± 4.2	19.2 ± 4.4		14.2 ± 4.5
CDR‐global, median (0–1)	0	0.5		0.5
CDR‐SOB, median (0–9)	0	0.5		3.0
NPI‐Q 12 domains (0–10)	1.0 ± 0.9	1.6 ± 1.7		2.3 ± 2.1
Severity (1–17)	1.1 ± 1.4	2.2 ± 2.6		3.2 ± 3.3
Caregiver distress (0–12)	1.0 ± 1.3	2.2 ± 3.3		2.9 ± 2.9
TICS‐Thai (9–39)	31.9 ± 4.7	28.2 ± 6.0		22.4 ± 6.5
Mini MoCA‐Thai (1–15)	11.3 ± 2.0	9.4 ± 2.3		5.9 ± 2.3

Abbreviations: CN = cognitively normal, MCI = mild cognitive impairment, IADL = instrumental activities of daily living, MMSE‐2 = mini‐mental state examination, second edition, MoCA = montreal cognitive assessment, CDR‐global = clinical dementia rating‐ global, CDR‐SOB = clinical dementia rating—sum of boxes, NPI‐Q = neuropsychiatric inventory questionnaire, TICS = telephone interview for cognitive status.

### 3.2 Validation Results

Table 2 presents Pearson correlation analyses between the TICS and Mini MoCA, as well as their correlations with MMSE‐2, MoCA, CDR‐Global, and CDR‐SOB scores. The TICS demonstrated a strong correlation with MMSE‐2 (*r* = 0.79, *p* < 0.001), while the Mini MoCA showed a similarly strong correlation with MoCA (*r* = 0.77, *p* < 0.001). Both the TICS and Mini MoCA were significantly correlated with global cognitive and functional measures.

**TABLE 2:** Pearson's correlation of TICS and Mini MoCA with MMSE‐2, MoCA, CDR‐Global, and CDR‐SOB.

Test	TICS	Mini MoCA	MMSE‐2	MoCA	CDR‐global	CDR‐SOB
TICS	1	—	—	—	—	—
Mini MoCA	0.78	1	—	—	—	—
MMSE‐2	0.79	0.74	1	—	—	—
MoCA	0.81	0.77	0.80	1	—	—
CDR‐Global	−0.56	−0.65	−0.59	−0.58	1	—
CDR‐SOB	−0.58	−0.71	−0.68	−0.59	0.74	1

*Notes*: All Pearson correlation coefficients were statistically significant at *p* < 0.001 (two‐tailed).

Abbreviations: TICS = telephone interview for cognitive status, MoCA = montreal cognitive assessment, MMSE‐2 = mini‐mental state examination, second edition, CDR‐global = clinical dementia rating‐ global, CDR‐SOB = clinical dementia rating—sum of boxes.

Subgroup analyses were conducted by age group (60–69, 70–79, ≥ 80 years) and by education level (<6, 7–12, > 12 years). As shown in Table X, both TICS and Mini MoCA declined with increasing age and increased with higher years of education. Correlation analyses supported these patterns, demonstrating significant negative correlations between age and both TICS (*r* = –0.267, *p* = 0.001) and Mini MoCA (*r* = –0.169, *p* = 0.043). Conversely, years of education showed significant positive correlations with TICS (*r* = 0.521, *p* < 0.001) and Mini MoCA (*r* = 0.379, *p* < 0.001), indicating better performance among individuals with higher educational attainment. (Table 3)

**TABLE 3:** Age and education stratified performance.

Test	Age, years (ranges)			Education, years (ranges)		
	60–70	70–80	80–90	< 6	7–12	> 12
TICS	32.1 ± 3.5	27.5 ± 7.1	24.9 ± 7.0	21.2 ± 5.8	27.4 ± 6.8	30.5 ± 5.6
Mini MoCA	10.3 ± 2.5	8.8 ± 3.2	8.2 ± 3.3	7.0 ± 3.0	8.5 ± 3.0	9.9 ± 2.9

TICS = telephone interview for cognitive status, Mini MoCA = mini montreal cognitive assessment.

Table 4 reports the sensitivity and specificity of the TICS and Mini MoCA in discriminating CN from dementia. The TICS demonstrated a sensitivity of 81.3% and specificity of 81.2% at an optimal cutoff score of 28/29 (AUC = 0.883). The Mini MoCA showed a sensitivity of 87.5% and specificity of 89.6% at a cutoff score of 8/9 (AUC = 0.958).

**TABLE 4:** Sensitivity, specificity, PPV, NPV, +LR, and −LR of the TICS and Mini MoCA in discriminating dementia and MCI from CN individuals, using DSM‐5 and CDR criteria for diagnosis.

TICS score	Sensitivity	Specificity	PPV	NPV	+LR	–LR
CN vs. dementia (AUC = 0.883, 95%CI = 0.82–0.95)					—	—
26/27	0.688	0.896	0.869	0.742	6.62	0.35
27/28	0.750	0.854	0.837	0.774	5.14	0.29
28/29	0.813	0.812	0.812	0.813	4.32	0.23
29/30	0.854	0.750	0.774	0.837	3.42	0.19
30/31	0.875	0.687	0.737	0.846	2.80	0.18
CN vs. MCI (AUC = 0.693, 95%CI = 0.59–0.80)					—	—
29/30	0.511	0.750	0.671	0.605	2.04	0.65
30/31	0.596	0.687	0.656	0.630	1.90	0.59
31/32	0.723	0.583	0.634	0.678	1.73	0.48
32/33	0.787	0.521	0.622	0.710	1.64	0.41
33/34	0.787	0.437	0.583	0.672	1.40	0.49
**Mini MoCA score**	**Sensitivity**	**Specificity**	**PPV**	**NPV**	**+LR**	**–LR**
CN vs. dementia (AUC = 0.958, 95%CI = 0.92–0.99)					—	—
6/7	0.583	0.979	0.965	0.701	27.76	0.43
7/8	0.750	0.979	0.973	0.797	35.71	0.26
8/9	0.875	0.896	0.894	0.878	8.41	0.14
9/10	0.917	0.833	0.846	0.909	5.49	0.10
10/11	0.979	0.687	0.758	0.970	3.13	0.03
CN vs. MCI (AUC = 0.755, 95%CI = 0.66–0.85)					—	—
8/9	0.362	0.896	0.777	0.584	3.48	0.71
9/10	0.489	0.833	0.745	0.620	2.93	0.61
10/11	0.723	0.687	0.698	0.713	2.31	0.40
11/12	0.872	0.500	0.636	0.796	1.74	0.26
12/13	0.915	0.250	0.550	0.746	1.22	0.34

Abbreviations: CN = cognitively normal, MCI = mild cognitive impairment, TICS = telephone interview for cognitive status, Mini MoCA = mini montreal cognitive assessment, CDR = clinical dementia rating, PPV = positive predictive value, NPV = negative predictive value, LR: likelihood ratio, AUC: area under the curve.

For distinguishing CN from MCI, the TICS yielded a sensitivity of 72.3% and specificity of 58.3% (cutoff 31/32; AUC = 0.693), while the Mini MoCA demonstrated a sensitivity of 72.3% and specificity of 68.7% (cutoff 10/11; AUC = 0.755).

Test‐retest and inter‐rater reliability were excellent, with intra‐class correlation coefficients of 0.933 and 0.995 for the TICS, and 0.918 and 0.998 for the Mini MoCA. The average administration time was 8.7 ± 1.9 min for the TICS and 4.4 ± 1.0 min for the Mini MoCA.

### 3.3 Comparison of TICS and Mini MoCA

**FIGURE 2:**
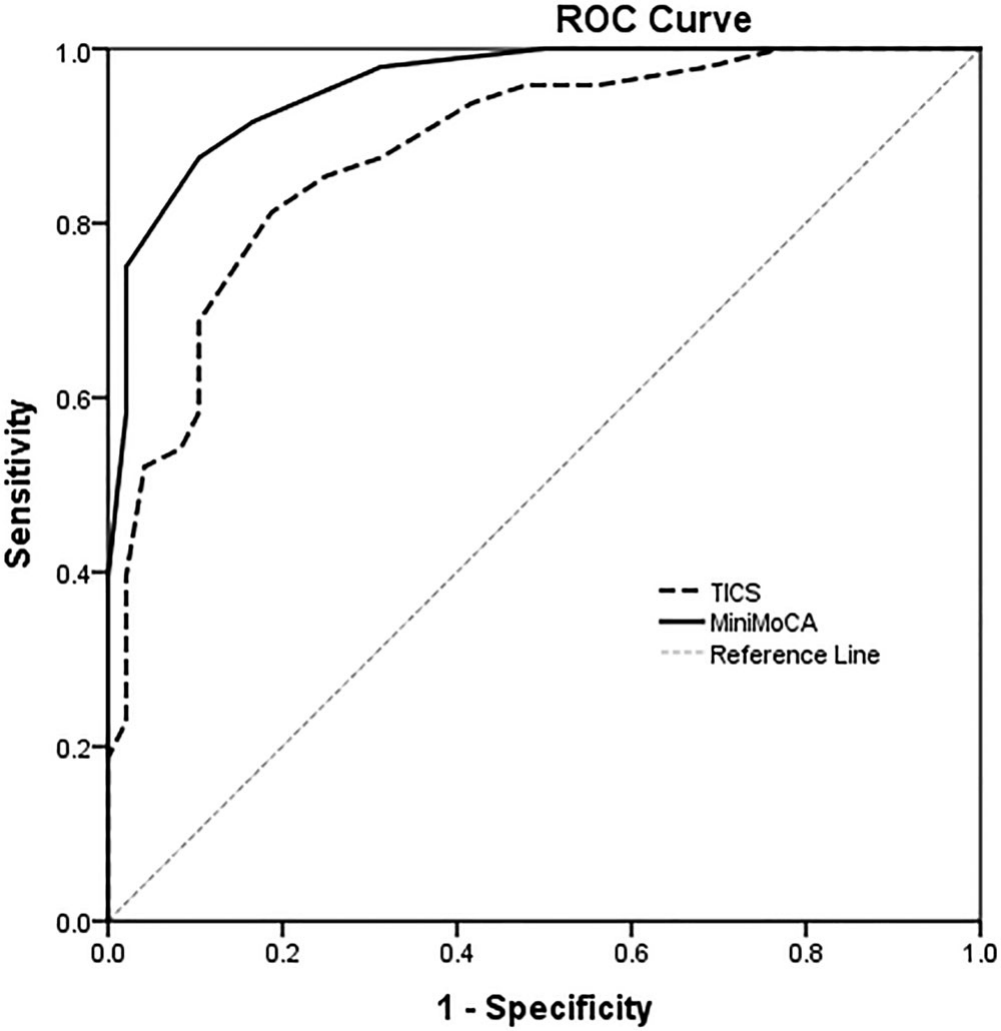
AUC for TICS and Mini MoCA in Detecting Dementia Defined by DSM‐5 Criteria and CDR.

To evaluate the comparative performance of the TICS and Mini MoCA as cognitive screening tools, ROC curve analyses were conducted in a sample of 149 participants. For the discrimination between CN individuals and those with dementia, both instruments demonstrated high diagnostic accuracy. The Mini MoCA showed slightly superior performance, with an AUC of 0.958 (95% CI: 0.92–0.99), compared to an AUC of 0.883 (95% CI: 0.82–0.95) for the TICS (Figure 2).

**FIGURE 3:**
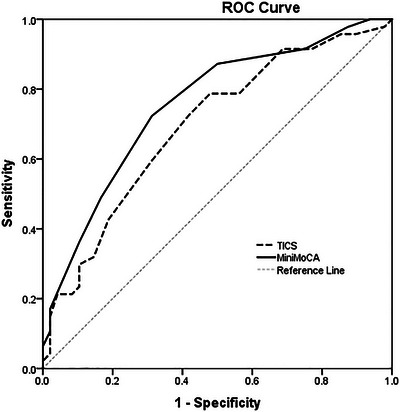
AUC for TICS and Mini MoCA in Detecting MCI Defined by DSM‐5 Criteria and CDR.

Similarly, for differentiating CN individuals from those with MCI, both instruments exhibited good discriminatory ability. Again, the Mini MoCA outperformed the TICS, with an AUC of 0.755 (95% CI: 0.66–0.85) versus 0.693 (95% CI: 0.59–0.80) for the TICS (Figure 3).

## 4 Discussion

This study provides the first validation of the Thai versions of the TICS‐Thai and the Mini MoCA‐Thai for detecting cognitive impairment among older Thai adults. Both instruments demonstrated excellent psychometric properties, (McDicken et al. 2019, Pendlebury et al. 2013; Wong et al. 2015) including strong test‐retest and inter‐rater reliability, and significant correlations with established cognitive and functional measures. These findings support their reliability and reproducibility for use in clinical practice and longitudinal cognitive monitoring (Seo et al. 2011; Baccaro et al. 2015; Georgakis et al. 2017).

Both tools showed good diagnostic accuracy in distinguishing CN individuals from those with MCI and dementia. Consistent with previous international studies, the sensitivity and specificity were higher for detecting dementia than for MCI (McDicken et al. 2019; Zietemann et al. 2017). The Mini MoCA‐Thai performed slightly better than the TICS‐Thai in identifying MCI, with higher AUC, sensitivity, and specificity values, aligning with earlier validation work of short‐form MoCA protocols. Importantly, our study extends the evidence base by being the first to evaluate Mini MoCA specifically for MCI detection, whereas most prior studies focused on dementia screening (Wong et al. 2015; Dong et al. 2016; Dujardin et al. 2021; Feng et al. 2021).

Cross‐cultural comparisons revealed that the optimal Mini MoCA‐Thai cutoff for dementia was slightly lower than that reported in a French validation study (Dujardin et al. 2021), potentially reflecting cultural and linguistic factors, diagnostic criteria (DSM‐5 vs. clinical consensus), and differences in participant characteristics. In contrast, the TICS‐Thai cutoff was higher than those reported in Korean (Seo et al. 2011) and Greek (Georgakis et al. 2017) studies, likely due to the relatively higher education level in our sample (mean 11.8 ± 5.3 years). Education is a known factor affecting cognitive test performance, particularly on language‐based and orientation tasks (Castanho et al. 2014; Georgakis et al. 2017; Wong et al. 2015).

Based on our ROC analyses, we propose the following preliminary single cut points for Thai adults aged 60 years and older. For the TICS‐Thai, a cut point of ≤ 28 provided the best discrimination for dementia, while a cut point of ≤ 31 performed optimally for identifying individuals with MCI. For the Mini MoCA‐Thai, a threshold of ≤ 8 demonstrated excellent discrimination for dementia, and ≤ 10 showed the strongest performance for detecting MCI. These thresholds may serve as practical clinical guidelines for cognitive screening in Thai older adults.

Compared to prior studies that used telephone versions of the full MoCA (T‐MoCA) primarily in post‐stroke settings, this study is among the first to compare TICS and Mini MoCA directly within the same population using standardized administration and rigorous diagnostic classification based on DSM‐5 and CDR criteria. Our findings are also consistent with reviews indicating that short‐form MoCA maintain high sensitivity and are particularly useful for rapid screening in time‐limited or remote settings (McDicken et al. 2019; Pendlebury et al. 2013).

Despite the limitations of telephone‐based tools, such as the absence of visuospatial tasks (Castanho et al. 2014; Pendlebury et al. 2013) both TICS and Mini MoCA capture essential domains (e.g., memory, attention, executive function) that are sensitive to early cognitive decline. (Wong et al. 2015; Dujardin et al. 2021) Furthermore, their verbal format makes them suitable for populations with visual impairments or limited access to in‐person care, which is particularly relevant in rural Thai settings. While the relatively high education level of our sample may limit generalizability, prior evidence suggests that both instruments remain valid across diverse educational backgrounds when culturally adapted. (Seo et al. 2011; Georgakis et al. 2017)

We acknowledge that participants who are able to attend a tertiary‐care, urban geriatric clinic are more likely to have higher educational attainment and better overall functioning. Although our sample had relatively high education, approximately 24.2% of participants had only primary‐level education, indicating some diversity in educational background. Nevertheless, further studies in rural populations will be important to evaluate the applicability of these tools in lower‐education community settings.

In addition to these considerations, another available telephone‐administered option is the telephone/blind MoCA. (Pendlebury et al. 2013) However, we did not include this measure in the present study for two reasons. First, a validated Thai version of the Telephone/Blind MoCA is not currently available. Second, its administration time (approximately 10 min) is considerably longer than that of the Mini MoCA (approximately 5 min), making it less practical for rapid screening or use in high‐volume clinical settings. (Wong et al. 2015) For these reasons, the Mini MoCA‐Thai was selected as the more feasible telephone‐based screening tool for this study.

This study also highlights future directions for expanding accessibility. Excluding individuals with sensory or physical impairments, though consistent with earlier protocols (Castanho et al. 2014), suggests a need to explore adaptations such as video‐assisted or caregiver‐supported formats to improve inclusivity.

This study has a few limitations. First, although 24.2% of participants in this study had low educational attainment, the overall relatively high education level of our sample—recruited from a tertiary‐care, urban geriatric clinic—may limit the generalizability of the findings to older adults in rural or lower‐education settings. Second, individuals with hearing impairment, severe sensory deficits, or communication limitations were excluded, which may restrict applicability in real‐world clinical contexts where such impairments are common. However, these limitations represent foreseeable and largely unavoidable challenges inherent to telephone‐based cognitive assessment. Future research in more diverse populations, including rural communities, will be essential to further validate the TICS‐Thai and Mini MoCA‐Thai across broader clinical settings. Moreover, generalizability to individuals with hearing, sensory, or communication limitations should be interpreted with caution.

## 5 Conclusions

The TICS‐Thai and Mini MoCA‐Thai are valid, brief, and reliable tools for cognitive screening in older Thai adults. Their feasibility for telephone administration makes them valuable for community‐based screening, epidemiological surveys, and telehealth applications. Mini MoCA‐Thai, in particular, shows promise as a rapid screening tool for early cognitive decline. These tools support Thailand's national strategy for aging care and may contribute to improved detection and management of dementia and MCI in real‐world settings.

## Author Contributions

**Pasa Sukson**: conceptualization, methodology, formal analysis, investigation, data curation, visualization, writing – original draft, writing – review and editing, project administration. **Weerasak Muangpaisan**: conceptualization, methodology, formal analysis, investigation, data curation, writing – original draft, writing – review and editing, project administration, funding acquisition, supervision. **Ananya Treewisut**: Methodology, Investigation, Writing – Review and Editing. **Supakorn Chansaengpetch**: Methodology, Investigation, Writing – Review and Editing. **Angkana Jongsawadipatana**: Methodology, Investigation, Writing – Review and Editing. **Pitiporn Siritipakorn**: Methodology, Investigation, Writing – Review and Editing. **Jirawit Wong‐ekkabut**: Methodology, Investigation, Writing – Review and Editing. **Somboon Intalapaporn**: Conceptualization, Methodology, Writing – Review and Editing, Funding acquisition.

## Funding

This study was supported by the Siriraj Research Development Fund, Faculty of Medicine Siriraj Hospital, Mahidol University, Bangkok, Thailand. The funder had no role in the interpretation of the data or the conclusions drawn from the study.

## Ethics Statement

The research protocol and all study procedures were approved by the Siriraj Institutional Review Board, Faculty of Medicine Siriraj Hospital, Mahidol University, Bangkok, Thailand.

## Consent

Written informed consent was obtained from all participants or their legal representatives prior to enrollment.

## Conflicts of Interest

The authors declare no conflicts of interest.

## Clinical Trial Registration

Not applicable

## Permission to Reproduce Material from Other Sources

All instruments used in this study were authorized by the copyright holders.

## Data Availability

The data that support the findings of this study are available from the corresponding author upon reasonable request.
